# RNA *N* ^6^‐methyladenosine modification in the lethal teamwork of cancer stem cells and the tumor immune microenvironment: Current landscape and therapeutic potential

**DOI:** 10.1002/ctm2.525

**Published:** 2021-09-26

**Authors:** Zhihui Zhang, Chaoqi Zhang, Yuejun Luo, Guochao Zhang, Peng Wu, Nan Sun, Jie He

**Affiliations:** ^1^ Department of Thoracic Surgery, National Cancer Center/National Clinical Research Center for Cancer/Cancer Hospital Chinese Academy of Medical Sciences and Peking Union Medical College Beijing China

**Keywords:** cancer stem cells, immune checkpoint, immunotherapy, m^6^A modification, metastasis, recurrence, therapeutic resistance, tumor immune microenvironment, PD‐1, PD‐L1

## Abstract

*N*^6^‐methyladenosine (m^6^A), the newest and most prevalent layer of internal epigenetic modification in eukaryotic mRNA, has been demonstrated to play a critical role in cancer biology. Increasing evidence has highlighted that the interaction between cancer stem cells (CSCs) and the tumor immune microenvironment (TIME) is the root cause of tumorigenesis, metastasis, therapy resistance, and recurrence. In recent studies, the m^6^A modification has been tightly linked to this CSC‐TIME interplay, participating in the regulation of CSCs and TIME remolding. Interestingly, the m^6^A modification has also been identified as a novel decisive factor in the efficacy of immunotherapies—particularly anti‐PD‐1/PD‐L1 monotherapies—by changing the plasticity of the TIME. Given the functional importance of the m^6^A modification in the crosstalk between CSCs and the TIME, targeting m^6^A regulators will open new avenues to overcome therapeutic resistance, especially for immune checkpoint‐based immunotherapy. In the present review, we summarize the current landscape of m^6^A modifications in CSCs and the TIME, and also prospect the underling role of m^6^A modifications at the crossroads of CSCs and the TIME for the first time. Additionally, to provide the possibility of modulating m^6^A modifications as an emerging therapeutic strategy, we also explore the burgeoning inhibitors and technologies targeting m^6^A regulators. Lastly, considering recent advances in m^6^A‐seq technologies and cancer drug development, we propose the future directions of m^6^A modification in clinical applications, which may not only help to improve individualized monitoring and therapy but also provide enhanced and durable responses in patients with insensitive tumors.

## Graphical abstract

In this review, we evaluate the current landscape of m6A modification in the lethal teamwork of CSCs and the TIME for the first time. In addition, we summarize the existing inhibitors targeting m^6^A regulators and discuss the potential of modulating m^6^A modification as a therapeutic strategy. Moreover, we propose the future directions of using m^6^A modification in cancer diagnosis and treatment, as well as providing an overview of the emerging approaches to m^6^A‐targeted immunotherapy. We believe that this review will provide novel insight into the role of m^6^A modification in cancer‐specific basic and translational medicine.

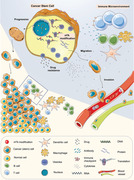

## 1 BACKGROUND

Since RNA modifications were first reported in 1951,^1^, ^2^ more than 150 types have been discovered owing to advances in high‐throughput sequencing technology.^3^ Of these, *N* ^6^‐methyladenosine (m^6^A) is recognized as the most essential and widespread type of modification in eukaryotic mRNAs and noncoding RNAs.^4^ A lack of advanced sequencing technologies has prevented any major breakthroughs in this field over past decades; however, with the successive discoveries of m^6^A regulatory components, the importance and function of the m^6^A modification has gradually been revealed. High‐throughput sequencing technologies have identified that m^6^A modification sites are often enriched in the coding sequence, 3’ untranslated region (3’UTR), and in the vicinity of stop codons.^1^, ^5^, ^6^ As with DNA and protein modifications, RNA m^6^A modification is a dynamic, reversible, and multilayered process that alters target gene expression based on the three types of m^6^A regulators (methyltransferases, demethylases, and binding proteins).^7^, ^8^ To date, numerous studies have demonstrated its involvement in various physiological and pathological processes, most notably in tumorigenesis.^9^

Increasing evidence suggests that the m^6^A modification plays a nonnegligible role in the evolution and progression of multiple tumors. It is noteworthy that a number of studies have found that m^6^A is engaged in the maintenance and modulation of the stemness property of cancer stem cells (CSCs).^10^, ^11^, ^12^ CSCs are a small subpopulation of tumor cells that possess self‐renewal and clonal tumor initiation potential, and are often regarded as one of the sources of tumor relapse, metastasis, and therapeutic resistance.^13^ Meanwhile, the tumor immune microenvironment (TIME), the lethal synthesis partner of CSCs, unites CSCs to form an ecological system for the acceleration of tumor progression.^14^, ^15^, ^16^ From the perspective of the seed and soil hypothesis, CSCs can be likened to the most tenacious intrinsic seed, while the TIME tends to represent the fertile soil conductive to the growth and survival of CSCs, which collude to promote a more malignant tumor phenotype with stronger metastatic and invasive capacity.^17^, ^18^ Theoretically, CSCs are able to remodel the TIME but are also inversely affected by signals originating from it.^15^, ^19^ Notably, it has also been reported that both CSCs and the TIME are intimately associated with resistance to the most promising immunotherapy, in particular immune checkpoint blockade therapy, and disruption of the CSC‐TIME interplay is a critical step in reducing resistance propensity and enhancing antitumor activity.^20^, ^21^, ^22^, ^23^ Intriguingly, m^6^A modification not only actively participates in the remodeling of TIME processes but also appears to be involved in the crosstalk between CSCs and the TIME during various immune responses and hypoxia‐related reactions.^24^, ^25^, ^26^, ^27^ It is reasonable to infer that m^6^A may be a pivotal factor in this deadly teamwork.

To date, the function of the m^6^A modification in the interaction between CSCs and the TIME remains under active investigation. In the present review, we provide a novel perspective on the role of the m^6^A regulatory network with respect to CSCs and the TIME. Additionally, we discuss the relevant molecular mechanisms and potential therapeutic strategies based on m^6^A modification, providing an overview of targeting the crosstalk between CSCs and the TIME for cancer therapies.

## 2 THE REGULATORY COMPONENTS OF RNA m^6^A MODIFICATION

**FIGURE 1:**
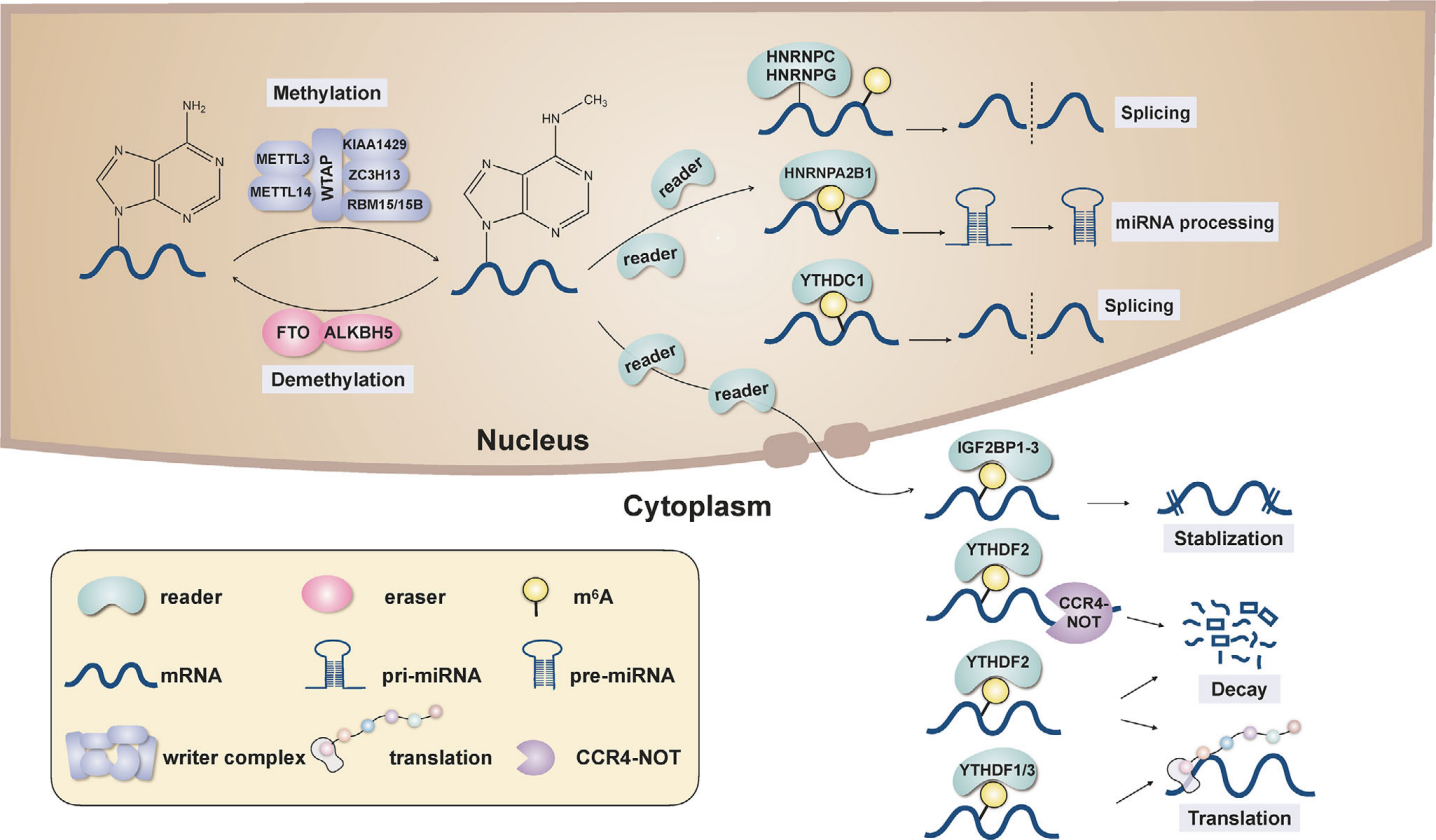
The cellular m^6^A machinery. m^6^A modification is a dynamic, reversible, and multilayered process. The writer complex has been identified as METTL3, METTL14, WATP, RBM15/15B, KIAA1429, and ZC3H13, which adds m^6^A methylation on target RNAs. The two erasers, FTO and ALKBH5, remove the m^6^A methylation from target RNAs. m^6^A is recognized by diverse readers, mainly HNRNPC, HNRNPG, HNRNBPA2B1, YTHDC1/2, and YTHDF1/2/3, which mediate various posttranscriptional processes including mRNA export, splicing, stabilization, decay, and translation, in addition to miRNA processing.

Similar to DNA and protein modifications, RNA m^6^A modification is a dynamic, reversible, and multilayered process that is determined by m^6^A‐specialized methyltransferases (writers), demethylases (erasers), and binding proteins (readers)^28^ (Figure 1). Owing to significant developments in the field of epitranscriptomics, a large number of writers, erasers, and readers have been identified.

### 2.1 m^6^A methyltransferases/writers

m^6^A writers are capable of adding m^6^A to RNA, which is regarded as the installation of the m^6^A modification, and often form a multisubunit complex to exert their effects, which includes methyltransferase‐like 3 (METTL13), methyltransferase‐like 14 (METTL14), wilms tumor 1‐associated protein (WTAP), vir‐like m^6^A methyltransferase associated (VIRMA/KIAA1429), RNA‐binding motif protein 15/15B (RBM15/15B), and zinc finger CCCH‐type containing 13 (ZC3H13). METTL3 and METTL14 are the core subunits of this methyltransferase complex, with METTL3 playing a leading enzyme‐catalyzing role and METTL14 being less enzymatically efficient. In addition, METTL14 also functions to stabilize METTL3 and recognize the target RNA.^29^ Despite lacking catalytic function, WTAP facilitates the localization of the METTL3/14‐METTL14 heterodimer to nuclear speckles.^30^ The function of ZC3H13 is to maintain and enhance the nuclear localization of the writer complex.^31^ KIAA1429 and RBM15/15B are responsible for ensuring that this complex is recruited in a specific region to exert catalytic action.^32^, ^33^, ^34^ In addition to this classical writer complex, other m^6^A methyltransferases have been consecutively discovered: methyltransferase‐like 5 (METTL5), methyltransferase‐like 16 (METTL16), zinc finger CCHC‐type containing 4 (ZCCHC4), phosphorylated CTD interacting factor 1 (PCIF1), and NOP2/Sun RNA methyltransferase 2 (NSun2). PCIF1 performs m^6^A methylation on 2‐*O*‐methylated adenine located at the 5’ end of mRNAs.^35^ With the exception of PCIF1, these writers are involved in the m^6^A modification of noncoding RNAs. In particular, METTL5 and ZCCHC4 are responsible for adding m^6^A on 18S and 28S ribosomal RNAs.^36^, ^37^ METTL16 is specifically engaged in the m^6^A modification of U6 small nuclear RNAs and also regulates the expression of MAT2A.^38^ Nsun2 actively participates in the regulation of m^6^A modification of tRNAs.^39^ Further research will reveal the more detailed functions of these writers.

**HIGHLIGHTS**

1. The teamwork of cancer stem cells (CSCs) and the tumor immune microenvironment (TIME) is the root cause of tumor progression;
2. The m^6^A modification is participated in the CSC−TIME interplay, and the interaction between m^6^A and CSC−TIME was overviewed for the first time;
3. This will provide novel insight into the role of m^6^A in cancer‐specific basic and translational medicine.

### 2.2 m^6^A demethylases/erasers

Erasers ensure that the m^6^A modification is a dynamic and reversible process. Currently, there are two predominant m^6^A demethylases, fat mass and obesity associated protein (FTO) and AlkB homolog 5 (ALKBH5), which belong to the ALKB family of dependent dioxygenases and collaborate to balance the m^6^A levels in the transcriptome by abrogating m^6^A modification of RNAs.^40^, ^41^, ^42^ However, these two erasers work in different ways, with FTO sequentially converting m^6^A to *N* ^6^‐hydroxymethyladenosine (hm^6^A), *N* ^6^‐formyladenosine (f^6^A), and adenosine, while ALKBH5 removes m^6^A in a straightforward manner.^42^, ^43^ To a large extent, these erasers select target RNAs to perform demethylation based on the structure and conformation elicited by m^6^A.^44^ Additionally, recent studies have shown that AlkB homolog 3 (ALKBH3) is also an emerging eraser of m^6^A modifications but acts preferentially on tRNAs.^45^

### 2.3 m^6^A binding proteins/readers

The mechanism by which m^6^A truly exerts its biological effects is through the recruitment of relevant binding proteins (readers). At present, the YT521‐B homology (YTH) domain family proteins (YTH domain‐containing proteins 1 [YTHDC1], YTH domain‐containing proteins 2 [YTHDC2], YTH N6‐methyladenosine RNA binding protein 1 [YTHDF1], YTH N6‐methyladenosine RNA binding protein 2 [YTHDF2], and YTH N6‐methyladenosine RNA binding protein 3 [YTHDF3]) are the most well‐studied m^6^A readers and can be classified into three types based on location: nuclear YTHDC1, nucleocytoplasmic YTHDC2, and cytoplasmic YTHDF1, YTHDF2, and YTHDF3.^46^, ^47^, ^48^ The main functions of YTHDC1 include RNA splicing, X‐chromosome silencing mediation, and facilitation of m^6^A‐modified RNA export from the nucleus to the cytoplasm.^46^, ^49^ YTHDC2 is tightly linked to both translation and RNA degradation.^50^ YTHDF1 directly promotes the translation of m^6^A‐modified RNAs in cooperation with the translation machinery.^51^ The ability of YTHDF2 to trigger the decay of target RNAs is achieved by the direct recruitment of CCR4‐NOT adenosine complexes.^52^ Interestingly, YTHDF3 serves as a synergistic role, assisting YTHDF1 in enhancing translation and YTHDF2 in causing RNA degradation.^53^ However, recent study revealed that YTHDF2 may play a double‐faceted role in RNA stabilization, not only inducing RNA degradation, but also stabilizing some RNA transcripts.^54^ In addition to the YTH domain family, other m^6^A‐binding protein families have also been identified, such as the insulin‐like growth factor 2 mRNA‐binding proteins (IGF2BP1, IGF2BP2, and IGF2BP3) and the heterogeneous nuclear ribonucleoprotein (HNRNP) family (HNRNPC, HNRNPG, and HNRPA2B1). IGF2BP1‐3 are involved in the maintenance and enhancement of target RNA stability and storage by interacting with the typical consensus GG(m^6^A)CU sequence.^55^ Unlike the YTH domain family, HNRNPC and HNRNPG are recognized as indirect readers since they do not bind to m^6^A modification sites, but preferentially bind to emerging sites generated by changes in RNA structure caused by m^6^A modification. This process, in which m^6^A modification regulates RNA‐structure‐based accessibility of m^6^A readers to alter target RNA biological function, is known as the m^6^A switch mechanism. It is through this m^6^A switch mechanisms that HNRNPC and HNRNPG affect the abundance and splicing of target RNAs.^56^, ^57^ In addition to being an indirect m^6^A reader, HNRNPA2B1 also actively modulates microRNA (miRNA) processing.^48^ Furthermore, other novel m^6^A readers have been discovered, including leucine‐rich pentatricopeptide repeat containing (LRPPRC), fragile X mental retardation protein (FMRP), eukaryotic initiation factor 3 (eIF3), and ATP binding cassette subfamily F member 1 (ABCF1).^58^, ^59^, ^60^, ^61^ These novel readers can also recognize m^6^A modifications to influence the fate of RNAs.

### 2.4 m^6^A modification in CSCs

**FIGURE 2:**
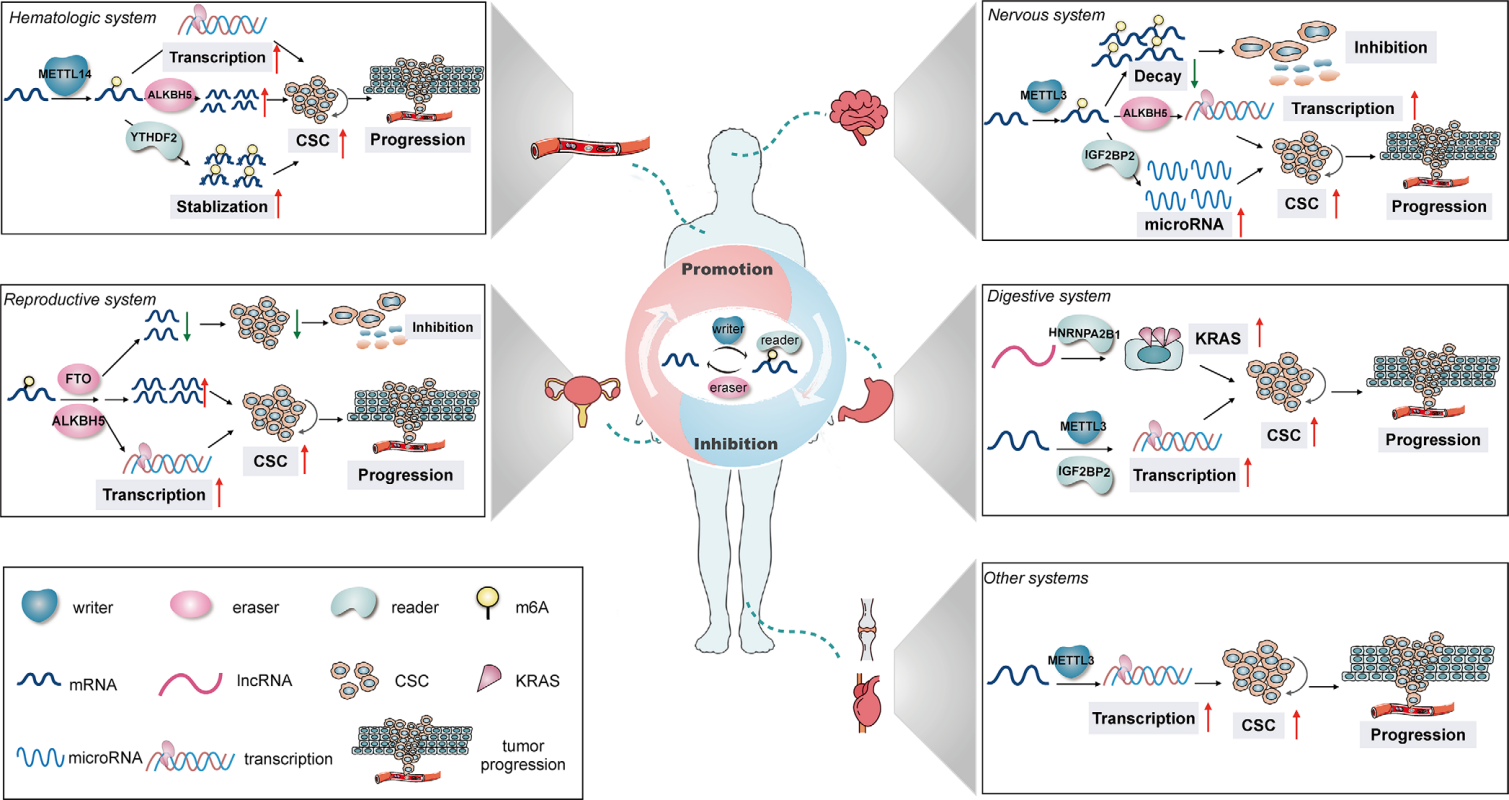
The relationship between m^6^A‐related regulators and various CSCs in different organ systems. The m^6^A‐related regulators are involved in various CSCs of different organ systems, including the nervous, digestive, reproductive, and hematologic systems. These regulators play a dual role in the modulation of various CSCs, promoting or inhibiting the stemness characteristics of CSCs by regulating the decay, splicing, stabilization, and translation of target RNAs.

A growing body of evidence shows that CSCs are inextricably linked to tumor initiation and development^62^, ^63^; therefore, it is believed that targeting CSC‐relevant mechanisms is the key foundation of anticancer treatment. Recently, it has been demonstrated that m^6^A modification contributes greatly to the pluripotency and differentiation of mammalian stem cells.^64^, ^65^ In particular, many representative pluripotent genes, such as nanog homeobox *(NANOG*), octamer binding transcription factor 3/4 (*OCT3/4*), kruppel like factor 4 (*KLF4*), and SRY‐Box transcription factor 2 (*SOX2)*, have been found to possess a large number of m^6^A modifications in their corresponding RNA transcripts.^66^, ^67^ Moreover, recent studies have shown that aberrant m^6^A deposition is also closely related to CSCs, which has been confirmed in a variety of malignancies.^10^, ^11^, ^68^ In the subsequent sections, we elaborate on the role of m^6^A modification in CSCs in different organ systems (Figure 2, Table 1).

**TABLE 1:** The relationship between m^6^A regulators and various CSCs

Cancer types	M6A regulators	Molecular axis	Function	Model system	Reference
GBM	METTL3	METTL3/SOX2	Promoting stemness of GSCs	In vivo	^71^
	METTL14	METTL3/METTL14/ADAM19	Inhibiting stemness of GSCs	In vitro and in vivo	^12^
	ALKBH5	ALKBH5/FOXM1	Promoting stemness of GSCs	In vitro and in vivo	^11^
	FTO		Promoting stemness of GSCs	In vivo	^12^
	IGF2BP2	IGF2BP2/Let‐7family	Promoting stemness of GSCs	In vitro and in vivo	^73^
		HMGA1	Promoting stemness of GSCs	In vivo	^74^
	YTHDF2	YTHDF2/MYC/IGFBP2	Promoting stemness of GSCs	In vitro and in vivo	^75^
PC	HNRNPA2B1	HNRNPA2B1/UCA1/KRAS	Promoting stemness of pancreatic CSCs	In vitro	^76^
	IGF2BP2	IGF2BP2/DANCR	Promoting stemness of pancreatic CSCs	In vitro and in vivo	^77^
CRC	METTL3	METTL3/SOX2	Promoting stemness of colorectal CSCs	In vitro and in vivo	^78^
	YTHDF1	YTHDF1/FZD9/WNT6/Wnt//β‐cantenin pathway	Promoting stemness of colorectal CSCs	In vitro and in vivo	^79^
HCC	YTHDF2	YTHDF2/OCT4	Promoting stemness of liver CSCs	In vitro and in vivo	^80^
	IGF2BP2	IGF2BP2/ROS production			^81^
BC	ALKBH5	ALKBH5/NANOG	Promoting stemness of BCSCs	In vitro and in vivo	^10^
OC	FTO	FTO/PDEC1/PDE4B/cAMP pathway	Inhibiting stemness of OCSCs	In vitro and in vivo	^85^
EC	ALKBH5	ALKBH5/SOX2	Promoting stemness of ECSCs	In vitro and in vivo	^25^
AML	METTL14	SPI1/METTL14/MYB/MYC	Promoting stemness of LSCs	In vitro and in vivo	^68^
	ALKBH5	ALKBH5/TACC3	Promoting stemness of LSCs	In vitro and in vivo	^88^
	FTO		Promoting stemness of LSCs	In vitro and in vivo	^89^, ^90^
	IGF2BP1	IGF2BP1/ALDH1A1/HOXB4/MYB	Promoting stemness of LSCs	In vitro and in vivo	^91^
	YTHDF2		Promoting stemness of LSCs	In vitro and in vivo	^92^
OS	METTL3	Pluripotency of stem cells/Wnt pathway	Promoting stemness of OSCs	In vitro	^94^
BCa	METTL3	METTL3/AFF4/SOX2/MYC	Promoting stemness of BCa stem cells	In vitro	^95^
cSCC	METTL3	METTL3/ΔNp6	Promoting stemness of cSCC stem cells	In vitro	^96^

m6A, N6‐methyladenosine; GBM, glioblastoma; PC, pancreatic cancer; CRC, colorectal cancer; HCC, hepatocellular carcinoma; BC, breast cancer; OC, ovarian cancer; EC, endometrial cancer; AML, acute myeloid leukemia; OS, osteosarcoma; BCa, bladder cancer; cSCC, cutaneous squamous cell carcinoma; GSCs, glioblastoma stem cells; CSCs, caner stem cells; BCSCs, breast cancer stem cells; OCSCs, ovarian cancer stem cells; ECSCs, endometrial cancer stem cells; LSCs, leukemia stem cells; OSCs, osteosarcoma cancer stem cells.

### 2.5 Nervous system

Glioblastoma (GBM) is the most prevalent and aggressive type of malignant nervous system tumors, with a high relapse potential and unfavorable prognosis.^69^, ^70^ The presence of glioblastoma stem cells (GSCs) is one of the root causes of this phenomenon and the primary concern related to GBM treatment strategies. Recent studies have demonstrated that m^6^A regulatory components, such as METTL3, ALKBH5, FTO, YTHDF2, and IGF2BP2, are involved in the modulation of GSCs.^11^, ^12^, ^71^, ^72^, ^73^, ^74^, ^75^ First, the m^6^A reader METTL3, a key mediator of GSCs, promotes the growth and self‐renewal of GSCs by stabilizing SOX2 mRNA.^71^ Downregulation of METTL3 has been shown to suppress the stemness features of GSCs and attenuate GBM invasiveness. Furthermore, high m^6^A modification levels enable reprogramming of GBM cells and transform non‐GSCs into GSCs. An in‐depth study uncovered that METTL3 protects serine and arginine rich splicing factors (SRSFs) mRNAs from nonsense‐mediated mRNA decay, which facilitates GBM development and progression^72^; however, with regard to the function of METTL3 in GBM, the opposite notion has been proposed.^12^ It was noted that overexpression of METTL3 inhibits the growth, self‐renewal, and frequency of GSCs by increasing the m^6^A abundance in the target mRNAs (eg, ADAM metallopeptidase domain 19 [ADAM19]), subsequently decreasing their expression. Further research is needed to explore these contradictory conclusions.

In addition, the eraser ALKBH5 is highly overexpressed in GSCs, predicting an unfavorable prognosis in patients with GBM. ALKBH5‐mediated demethylation of the transcription factor forkhead box M1 (FOXM1) results in elevated corresponding nascent transcripts and the subsequently detectable increased expression of mRNA and protein, which ultimately boosts GSC function and GBM germination. Moreover, knockdown of ALKBH5 has been shown to be effective in reducing the proliferative capacity of GSCs and weakening their stemness features.^11^ Furthermore, pharmacological inhibition of eraser FTO impedes GBM growth and tumor initiation while also prolonging the life expectancy of GSC‐engrafted mice.^12^

Moreover, two m^6^A binding proteins have been reported to participate in the regulation of GSCs. IGF2BP2 specifically binds to the let‐7 miRNA recognition sites of target transcripts to protect against let‐7 miRNA‐based splicing and silencing of these target genes, which is thought to increase the expression levels of corresponding mRNA and protein levels and subsequently induce and preserve GSCs specificity.^73^ Another study revealed the relationship between IGF2BP2 and GSCs in mesenchymal GBM, where IGF2BP2, DExH‐Box helicase 9 (DHX9), and HIF1A antisense RNA 2 (HIF1A‐AS2) can directly interact to stimulate the expression of target genes (high mobility group A1 [HMGA1]), eventually driving the GBM phenotype and enabling GSCs to acclimatize to hypoxic conditions.^74^ Similarly, YTHDF2 serves as an oncogenic trigger in GBM hierarchy, being upregulated in GSCs and supporting their stemness. This reader stabilizes transcripts of MYC and VEGFA in an m^6^A modification‐dependent manner, both of which subsequently interact with the downstream effector IGF2BP2 to establish a strong axis in GBM. The YTHDF2‐MYC‐IGF2BP2 axis may be a potential novel therapeutic target for GBM treatment.^75^

### 2.6 Digestive system

At present, it is understood that m^6^A regulators act as essential modulators of CSCs in three digestive malignancies: pancreatic cancer (PC), colorectal cancer (CRC), and hepatocellular carcinoma (HCC). First, HNRNPA2B1 has been reported to synergize with a long noncoding RNA urothelial cancer associated 1 (UCA1) to enhance KRAS expression and activity, ultimately facilitating CSCs properties and tumor growth in PC.^76^ In addition, IGF2BP2 stabilizes the long noncoding differentiation antagonizing nonprotein coding RNA (DANCR) in an m^6^A modification‐based manner, which work in concert to contribute to the stemness properties and progression in PC.^77^ Moreover, METTL3 collaborates with IGF2BP2 to increase m^6^A enrichment of SOX2 transcripts as well as extend their lifespan, maintaining and motivating the stemness of CRC cells.^78^ Researchers have also found that a high expression level of YTHDF1 promotes the tumorigenicity of CRC cells both in vivo and in vitro, while silencing YTHDF1 gives rise to a corresponding downregulation of classical CSC markers (CD44, CD133, OCT4, aldehyde dehydrogenase 1 [ALDH1], and leucine‐rich repeat‐containing G‐protein coupled receptor 5 [LGR5]), a smaller clonosphere, and slower tumor formation. Theoretically, suppression of YTHDF1 decreases frizzled class receptor 9 (FZD9) and Wnt family member 6 (WNT6) levels, ultimately limiting the stem cell‐related Wnt/β‐catenin signaling pathway.^79^ Several m^6^A regulators have been identified as playing pivotal roles in HCC CSCs regulation. YTHDF2 functions to regulate m^6^A methylation levels in the 5’UTR of OCT4 mRNA and influence the protein translation of OCT4 mRNA; therefore, the activity of CSCs can be enhanced or weakened by altering the expression level of YTHDF2. In vitro experiments have demonstrated that the loss of YTHDF2 lowers tumor burden and the likelihood of lung metastasis in HCC.^80^ Furthermore, another m^6^A binding protein IGF2BP2 enables to improve reactive oxygen species (ROS) production and induce genomic instability in HCC CSCs.^81^

### 2.7 Reproductive system

Breast cancer (BC) is the leading killer in women, with unparalleled morbidity and mortality rates.^82^ Similar to other tumors, aberrant m^6^A modification also promotes the strength of breast cancer stem cells (BCSCs).^10^ Upregulation of ALKBH5 increases m^6^A demethylation of NANOG, NANOG mRNA stability, and protein expression, ultimately boosting the BCSCs phenotype in BC. In vitro experiments have demonstrated that silencing ALKBH5 suppresses tumor formation and dramatically diminishes the proportion and function of BCSCs.^10^ Moreover, both ovarian and endometrial cancer (OC and EC) are serious diseases in women.^83^, ^84^ In OC, FTO plays a tumor suppressor role and impairs ovarian cancer stem cells (OCSCs) function when overexpressed. Owing to its demethylase activity, FTO destabilizes the mRNAs of two phosphodiesterase genes (phosphodiesterase 1C/4B [*PDEC1* and *PDE4B*]) to strengthen cyclic adenosine monophosphate (cAMP) signaling and dampen the stemness characteristics of OCSCs.^85^ Similarly, endometrial cancer stem cells (ECSCs) are also controlled by ALKBH5, the high expression of which mediates the SOX2 level through its enhanced demethylation capacity, triggering ECSCs initiation and stemness states.^25^

### 2.8 Hematologic system

Leukemia stem cells (LSCs), featured by an unparalleled self‐renewal and growth capacity, are believed to be the initial trigger of the emergence and development of this hematologic malignancy, as well as problems related to treatment resistance and recurrence.^86^, ^87^ Researchers have successively unraveled the connection between m^6^A modification and LSCs, elucidating that m^6^A regulation is indispensable for LSCs growth.

METTL14, an essential element of the m^6^A writer complex, has been found to be highly expressed in both normal hematopoietic stem cells (HSCs) and acute myeloid leukemia (AML) cells; however, its expression decreases when these cells begin to differentiate. MYB and MYC, playing significant roles in the differentiation and self‐renewal of AML cells, are the direct targets of METTL14. This m^6^A writer targets the posttranscriptional regulation of MYB and MYC based on m^6^A modification, while its own expression is controlled by SFI1 centrin binding protein (SFI1). This biological process constructed a novel signaling axis (SPI1‐METTL14‐MYB/MYC) in the AML that determines the fate and activity of LSCs.^68^ Notably, the small molecule inhibitor of METTL3 was effective to decrease the AML stem cells growth and propagating, also enabled to prolong the survival of multiple AML patients‐derived‐xenografts mouses models.^88^ ALKBH5 is also aberrantly elevated in AML, which often predicts poor survival in AML patients. Inhibition of ALKBH5 impairs the growth and self‐renewal of LSCs yet has little effect on normal HSCs. Mechanistically, it has been discovered that ALKBH5 directly modifies Transforming acidic coiled‐coil 3 (TACC3) in an m^6^A posttranscriptional manner to ensure its transcripts half‐life and high expression. Moreover, it was highlighted that ALKBH5 is a specific target for eradicating LSCs in AML.^89^ With respect to another identified m^6^A eraser FTO, both its pharmacological inhibition and knockdown can inhibit LSCs population growth and function, which also increases the toxic effect of T cells on LSCs.^90^, ^91^ The m^6^A binding proteins, IGF2BP1 and YTHDF2, have also been associated with LSCs. IGF2BP1 orchestrates LSCs phenotype and tumor‐initial property. Suppression of IGF2BP1, genetically and pharmacologically, decreases LSCs proliferation, increases LSCs differentiation, induces programmed death of LSCs, and enhances the sensitivity of LSCs to chemotherapy. The potential and underlying cause may be the ability of IGF2BP1 to directly modulate crucial regulators and stemness markers of HSCs, including Aldehyde dehydrogenase 1 family member A1 (ALDH1A1), Homeobox B4 (HOXB4), and MYB.^92^ Similarly, YTHDF2 is also an active participant in the transformation of AML, and inactivation of YTHDF2 prolongs the half‐life of transcripts with m^6^A modifications, which are mostly associated with LSCs. The absence of YTHDF2 renders LSCs more sensitive to TNF‐induced apoptotic signals. Interestingly, silencing of YTHDF2 promotes HSCs to a certain extent, but does not allow hematopoiesis to become out of control.^93^

## 3 OTHER SYSTEMS

Unsurprisingly, in other systems, recent literature has also uncovered the presence of m^6^A modifications in the regulation of various CSCs. Since related studies are still at a relatively early stage, we have included these different systems into the same section for elaboration.

METTL3 is perceived as an essential performer of m^6^A modifications in osteosarcoma (OS), bladder cancer (BCa), and cutaneous squamous cell carcinoma (cSCC), mediating the stemness properties of CSCs and tumor progression.^94^, ^95^, ^96^, ^97^ The expression of METTL3 is elevated in osteosarcoma stem cells (OSCs), causing the increased m^6^A modifications in OSCs as compared with non‐OSCs, which may be one of the principal reasons why OS is prone to chemoresistance and metastasis. Moreover, bioinformatics analysis has suggested that these variations in m^6^A enrichment are likely related to pluripotency of stem cells and the Wnt pathway.^95^ In BCa, the emergence and self‐renewal of CSCs is limited when METTL3 is depleted. Mechanistically, METTL3 controls the expression of AF4/FMR2 family member 4 (AFF4), and AFF4 upregulates the essential stemness genes *SOX2* and *MYC* by binding to their promoter regions.^96^ METTL3 deficiency has been proven to alter the expression of typical differentiation and undifferentiation markers (K10 and K14) in cSCC stem cells. Depletion of METTL3 dramatically restricts ΔNp63 expression, which consequently leads to poorer growth and tumorigenicity of cSCC stem cells.^97^ In addition, YTHDF1 has also been demonstrated to help tumor cells adapt to hypoxic conditions in lung cancer, while hypoxia‐related molecular events are known to facilitate and support CSCs development. This phenomenon suggests that YTHDF1 may govern CSCs in an indirect manner in lung cancer.^24^, ^98^

In summary, it can be clearly observed that m^6^A modification plays a key role in the emergence and development of CSCs in a variety of malignancies. The presence of CSCs is often considered the root source of tumor propagation and relapse, which in turn are also responsible for chemoradiotherapy resistance. Furthermore, chemotherapy drug transport and metabolism are also mediated by m^6^A modification, which is the first step in determining drug effectiveness against CSCs.^99^ Therefore, it is reasonable to assume that the biological process of m^6^A modification may be pivotal in eliminating CSCs and destroying tumors, providing a novel and potential therapeutic avenue for several cancers.

### 3.1 m^6^A modification in the TIME

**FIGURE 3:**
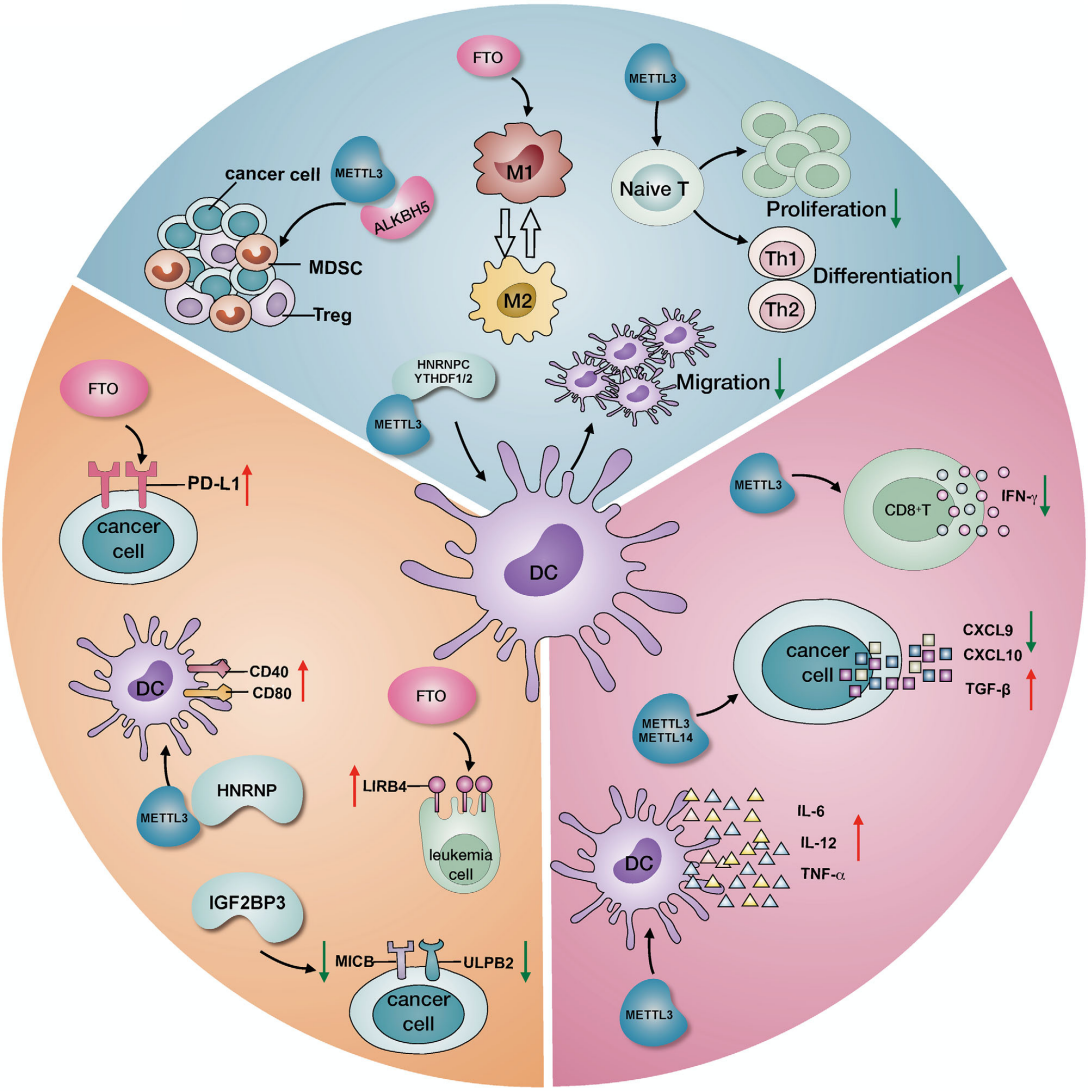
The m^6^A‐mediated TIME remodeling includes multiple aspects. m^6^A‐related regulators actively reprogram the TIME in various ways. For immune cells, these regulators are able to influence MDSC and Treg infiltration, M1 and M2 polarization, DC migration and function, and naïve T cell proliferation and differentiation. For immune checkpoints, PD‐L1, LIRB4, CD40, and CD80 are upregulated by certain m^6^A regulators, while MICB and ULPB2 are downregulated. For cytokine production, the secretion of IL6, IL12, TNF‐α, and TGF‐β is increased, but that of CXCL9, CXCL10, and IFN‐γ production is decreased.

With the recognition of cancer as a heterogeneous disease, the TIME can no longer be separated from tumorigenesis and progression.^100^ The TIME is a complex and systematic structure, analogous to fertile soil for tumor growth, featured by various immune cellular compositions, regulatory‐protein expression, and inflammatory cytokines.^18^, ^21^ Most importantly, the TIME status is the leading cause of the differential responses and outcomes in cancer patients receiving the same treatment, especially for multiple immunotherapies.^21^, ^101^ Therefore, explaining the diversity and complexity of the TIME is an indispensable step in enhancing the predictive capacity and clinical guidance of immunotherapy, which will benefit countless patients. It is becoming apparent that TIME remodeling includes various biological processes, such as immune cell infiltration, immune checkpoint protein expression, and cytokine production, in which the m^6^A modifications function as a critical mediator (Figure 3, Table 2).

**TABLE 2:** m6A‐mediated TIME remodeling in various aspects

TIME compositions		M6A regulators	Molecular mechanisms	Functions	Reference
Immune cells	DC	METTL3, HNRNPC	Regulating CD40 and CD80 expression	DC migration and function	^27^, ^102^
		YTHDF1, YTHDF2	Regulating lysosomal protease expression	CCR7‐mediated DC migration and DC‐based immunec responses	^103^
	Macrophage	FTO	Regulating STAT1 and PPAR‐γ mRNA degradation	M1 and M2 macrophages polarization	^105^
	Naïve T cell	METTL3	Regulating SOCS family mRNA degradation	naïve T cell proliferation and differentiation	^106^
	Treg	METTL3	Regulating SOCS family mRNA degradation	Tregs stability and suppressive function	^107^
		ALKBH5	Regulating MCT4 expression	Tregs infiltration	^26^
	MDSC	METTL3		MDSCs infiltration	^108^
		ALKBH5	Regulating MCT4 expression	MDSCs infiltration	^26^
Immune checkpoints	PD‐L1	FTO	Regulating IFN‐γ pathway	PD‐L1 expression	^110^
	LIRB4	FTO	Regulating mRNA degradation	LIRB4 expression	^88^
	ULPB2, MICB	IGF2BP3		ULPB2 and MICB expression and migration	^111^
	CD40, CD80	METTL3, HNRNPC		CD40 and CD80 expression	^27^, ^102^
Cytokines	IL6, IL12, and TNF‐α	METTL3	Regulating NF‐κB signaling	Production of IL6, IL12, and TNF‐α	^27^
	IFN‐γ, CXCL9, and CXCL10	METTL3, METTL14	Regulating IFN‐γ‐STAT1‐IRF1 signal pathway	Production of IFN‐γ, CXCL9, and CXCL10	^113^
	TGF‐β1	METTL3	Regulating TGF‐β1 mRNAs degradation and translation elongation	Production and activation of TGF‐β1	^115^

m^6^A, *N* ^6^‐methyladenosine; TIME, tumor immune microenvironment; DC, dendritic cell; CCR7, C‐C motif chemokine receptor 7; STAT1, signal transducer and activator of transcription 1; PPAR, peroxisome proliferators‐activated receptor; SOCS, suppressor of cytokine signaling; Treg, regulatory T cells; MCT4, monocarboxylate transporter 4; MDSC, myeloid‐derived suppressor cell; IFN‐γ, interferon gamma; LIRB4, leukocyte immunoglobulin like receptor B4; LIRB4, leukocyte immunoglobulin like receptor B4; MICB, MHC class I polypeptide‐related sequence B; IL‐6, interleukin 6; IL‐12, interleukin 12; TNF‐α, tumor necrosis factor alpha; NK‐κB, nuclear factor kappa B; CXCL9, C‐X‐C motif chemokine ligand 9; CXCL10, C‐X‐C motif chemokine ligand 10; TFG‐β1, transforming growth factor beta 1.

### 3.2 Immune cells

The TIME is broadly populated with various immune cells, and their composition, distribution, and function largely determine the TIME characteristics.^21^ Dendritic cell (DC)‐directed antigen presentation is the initial step in the activation of antitumor effects of specific immunity, while m^6^A modification influences DCs in many ways. METTL3, HNRNPC, YTHDF1, and YTHDF2 affect the maturation and phenotype of DCs, further disrupting their key immune functions. On one hand, both METTL3 and HNRNPC regulate DC maturation and function by altering the costimulatory molecular (CD40 and CD80) expression.^27^, ^102^ METTL3 has been found to promote translation of CD40 and CD80 mRNAs. In addition, loss of METTL3 in DCs decreases the transcription of TLR4 signaling adaptor Tirap, resulting in a diminished ability to activate T cells.^27^ On the other hand, YTHDF1 and YTHDF2 manipulate DC migration and function through completely different mechanisms. In DCs, YTHDF1 directly binds to lysosomal protease transcripts marked by m^6^A and promotes the translation of lysosomal cathepsins, rendering DCs incapable of neoantigen‐presentation and cross‐priming.^103^ CCR7‐mediated DC migration and DC‐based immune response pathways can also involve the presence of m^6^A modifications. The lnc‐RNA double PHD fingers 3 (Dpf3), which plays a pivotal role in these pathways, directly impedes hypoxia‐inducible factor 1‐alpha (HIF‐1α) activity and HIF‐1α‐dependent glycolytic metabolism, culminating in the inhibition of DC migration and inflammatory responses. Silencing of YTHDF2 further exacerbates this CCR7‐induced DC migration process and completely disables DCs by alleviating m^6^A modification‐based RNA degradation of lnc‐Dpf3.^104^ In addition to DCs, macrophage polarization is also fine‐tuned by m^6^A modification. Depletion of the m^6^A demethylase FTO not only inactivates the NF‐κB signaling pathway but also restricts the polarization of M1 and M2 macrophages owing to the accelerated degradation of signal transducer and activator of transcription 1 (STAT1) and peroxisome proliferators‐activated receptor‐γ (PPAR‐γ) mRNA marked by m^6^A.^105^ Furthermore, m^6^A methylation‐mediated mRNA decay disturbs homeostasis and activates naïve T cells and Tregs.^106^, ^107^ Downregulation of METTL3 results in a considerable decline in m^6^A‐dependent mRNA degradation of the suppressor of cytokine signaling (SOCS) family, which encode many STAT family repressor proteins. Consequently, these inhibitor proteins, such as rate‐limiting enzymes, restrain naïve T cell proliferation and differentiation mediated by IL‐7 signaling and also decrease IL‐2 signaling, disturbing the stability and suppressive function of Tregs.^106^, ^107^ There is no doubt that CD8+ T cells are also influenced by m^6^A regulators. In vivo experiments, both METTL3 and METTL14‐deficient tumors render higher infiltration of CD8+ T cells and enhanced secretion ability of cytokines than the controls.^108^ Meanwhile, YTHDF1‐knock out mice exhibit increased cross‐priming of CD8+ T cells by DCs as compared with WT mice.^103^ Interestingly, patients with low YTHDF1 expression have higher proportion of CD8+ T cells in the TIME.^103^

Moreover, the expression level of METTL3 is positively correlated with myeloid‐derived suppressor cells (MDSCs) infiltration in the TIME, which work together to create an immunosuppressive environment. Both METTL3 and MDSCs are independent factors for reduced survival in cervical cancer patients.^109^ Similarly, the m^6^A eraser ALKBH5 indirectly manipulates the splicing and expression of the target gene monocarboxylate transporter 4 (MCT4), a crucial lactate transporter, the expression of which is subject to m^6^A demethylation by ALKBH5. Inhibition of ALKBH5 by CRISPR or pharmacological molecules reduces MCT4 expression, leading to a dramatic decrease in lactate content of the TIME. A lower lactate concentration is accompanied by a smaller proportion of suppressive immune cells (Tregs and MDSCs) in the TIME, which notably harbor ALKBH5 deletions or mutations and typically predict sensitive responses and favorable efficacy of anti‐PD‐1 treatment in melanoma patients.^26^ Moreover, Li et al constructed a comprehensive m^6^A regulator‐based risk signature that implicates a strong relationship between m^6^A regulators and immune cell infiltration in the TIME. Based on bioinformatics analysis, they also prompted m^6^A may collaborate with the PI3K‐AKT‐mTOR signaling pathway to reprogram the TIME.^110^

### 3.3 Immune checkpoints

Conceivably, some critical immune checkpoints are also supervised by m^6^A regulators. In colon cancer cells, FTO overexpression causes a corresponding boost in PD‐L1 protein expression in an IFN‐γ‐dependent manner.^111^ In addition, FTO actively upregulates immune checkpoint LIRB4 in AML cells by reducing YTHDF2‐induced mRNA degradation. Blockade of FTO is an effective way to sensitize AML cells to T‐cell cytotoxicity by targeting leukocyte immunoglobulin like receptor B4 (LIRB4), which alleviates tumor immune evasion in AML to some degree.^90^ Further, the m^6^A binding protein IGF2BP3 also takes part in the modulation of tumor immune evasion, showing a remarkably higher expression level in tumor cells than in normal tissue. Powerfully oncogenic IGF2BP3 not only downregulates the expression of stress‐induced ligands (UL16 binding protein 2 [ULPB2] and MHC class I polypeptide‐related sequence B [MICB]) but also prohibits these proteins from trafficking to the cell surface. Moreover, NK cells fail to recognize and annihilate these ingenious tumor cells via the natural killer group 2 member D (NKG2D) receptor.^112^ Furthermore, both METTL3 and HNRNPC mediate the expression of the costimulatory molecules CD40 and CD80 in DCs. METTL3‐deficient DCs demonstrate lower CD40 and CD80 translation levels than METTL3‐wild type DCs. It has also been shown that METLL3 promotes CD40 and CD80 expression in DCs by increasing the translation efficiency of the corresponding mRNAs.^27^

Anti‐PD‐1/L1 treatment is perceived to be the most promising method of annihilating and eradicating malignancies,^113^ and researchers have gradually unveiled the roles of certain m^6^A regulators in anti‐PD‐1/L1 therapy. Intriguingly, the efficacy of anti‐PD‐1 is considerably augmented by simultaneous depletion of METTL3 and METTL14 in both colorectal cancer and melanoma. Mechanistically, the depletion of METTL3 and METTL14 may increase the infiltration and function of CD8+T cells.^108^ Similarly, the m^6^A eraser ALKBH5 indirectly affects responses to anti‐PD‐1 immunotherapy by creating a suppressive TIME. Conceivably, harboring ALKBH5 deletions or mutations typically predicts sensitive responses and a favorable efficacy of anti‐PD‐1 treatment in melanoma patients.^26^ In parallel, knockdown of FTO results in increased PD‐1 expression, which reverses melanoma resistance to anti‐PD‐1 therapy in preclinical experiments.^114^ Moreover, YTHDF1‐deficient mice are featured by more CD8+ T cells in TIME, tending to display preferable efficacy profiles and outcomes for PD‐L1 checkpoint blockade.^103^ Taken together, this evidence demonstrates that all aspects of m^6^A regulators are competent in modulating the immune responses to anti‐PD‐1/L1 treatment.

### 3.4 Cytokines

It has been reported that the cytokine production process is also controlled by m^6^A regulators‐mediated signaling pathways. METLL3 enhances NF‐κB signaling by controlling the levels of downstream effector molecules. MELLT3‐knockout DCs exhibit a dramatic decrease in production of the cytokines IL6, IL12, and TNF‐α following lipopolysaccharides (LPS) stimulation. Mechanistically, METTL3 deficiency causes a lower translation level of these cytokine mRNAs.^27^ Also, the type IFN‐β production raised by human cytomegalovirus or double‐stranded DNA was influenced by METTL14 and ALKBH5. Inhibiting the METTL14 genetic expression decreased the production and accumulation of type IFN‐β in the above process; however, inhibition of ALKBH5 obtained the opposite result.^115^ Previous studies have uncovered that these cytokines are positively involved in the accumulation and response of T cells and the subsequent antitumor effect.^116^, ^117^, ^118^, ^119^ Importantly, the combined targeting of IL6 with PD‐1/PD‐L1 and blocking of TNF and PD‐1 enables to increase the efficacy of PD‐1/PD‐L1 treatment, which significantly reduce tumor progression.^117^, ^118^, ^120^ Moreover, it has also been found that FTO is engaged in NF‐κB signaling, and inhibition of FTO leads to inactivation of this pathway.^105^ In colorectal cancer and melanoma, simultaneous depletion of METTL3 and METTL14 induces mass production of cytokines, including IFN‐γ, CXCL9, and CXCL10. The underlying mechanism is that suppression of METTL3 and METLL14 contributes to the stabilization of STAT1 and interferon regulatory factor 1 (IRF1) mRNAs, which orchestrate the IFN‐γ‐STAT1‐IRF1 signaling pathway in the TIME.^108^ Most strikingly, METTL3 has also been found to mediate the expression and secretion of TGF‐β1, which further modulates the TGF‐β‐induced epithelial‐mesenchymal transition (EMT) process of tumors. It has been suggested that the METTL3 expression level is negatively correlated with TGF‐β1 mRNA decay and translation elongation. In METTL3‐knockdown cancer cells, the half‐life, secretion, and activation of TGF‐β are inhibited, which contributes to the suppression of the biological process of EMT.^121^

To summarize, the m^6^A modification has shown tremendous potential in remodeling the TIME in various dimensions, including the function of multiple immune cells, cytokine secretion, and expression of immune checkpoint proteins. Therefore, it is believed that only the tip of the iceberg related to the role and mechanisms of the m^6^A modification in the reprograming of the TIME has been uncovered to date. Elucidation of the manner by which m^6^A modification remodels the TIME is not only essential for perceiving tumor biology but also provides a novel opportunity to explore the potential of this RNA regulation‐based therapy in cancer.

### 3.5 m^6^A modification: an underlying bridging between CSCs and the TIME

CSCs are notorious for being the root cause of tumor recurrence and resistance, which render current treatments ineffective in a vast number of patients. Moreover, the TIME is also a primary determinant of the efficacy of various therapies, especially immunotherapy. It has become apparent that CSCs and the TIME are never mutually exclusive in tumors but establish a deadly teamwork to facilitate tumor development and progression, serving as putative catalysts for each other. In the crosstalk between the CSC‐TIME, both intercellular contact and noncontact interactions occur. Taking the noncontact interactions as an example, tumor cells produce various cytokines to stimulate the expansion of suppressive immune cells, while these immune cells also secrete soluble factors to enhance the CSCs plasticity and the EMT process.^122^

Due to the rapid and unrestrained proliferation of tumor cells, hypoxia is a pervasive and prominent feature of the TIME. Hypoxia is indispensable for CSCs maintenance but also supports the acquisition of stemness characteristics in tumors. Strikingly, it has been reported that certain m^6^A regulators collaborate with HIF‐1α and HIF‐2α to promote the CSCs phenotype in various tumors.^10^, ^25^ Exposure to a hypoxic microenvironment profoundly stimulates the expression of HIFs and ALKBH5 in BC cells, which ultimately advances BCSCs stemness features and enrichment. During this biological process, HIFs may act as upstream regulators of ALKBH5‐mediated demethylation targeting the pluripotent gene *NANOG*, since changing the expression level of HIFs alters the activity of ALKBH5 accordingly. Dual regulation by HIFs and ALKBH5 gives rise to higher expression and lower degradation levels of target gene *NANOG*, ultimately increasing the percentage of BCSCs in BC.^10^ The same applies to ECSCs, in which suppression of HIFs also markedly decreases the protein expression level of ALKBH5 and thus diminishes its demethylation capacity. HIFs and ALKBH5 form a subtle collaboration to mediate the level of SOX2, which is the trigger for ECSCs initiation and development. This HIFs‐ALKBH5‐SOX2 axis endeavors to maintain the ECSCs phenotype and function in the TIME.^25^ These findings indicate that the m^6^A modification functions as a connector in the process of hypoxia‐induced stemness in ECSCs.

Meanwhile, the costimulatory and adhesion molecules also actively participate in the contact interaction in the CSC‐TIME connection, for instance, PD‐L1 signaling acts as an essential role to by promoting immune evasion and CSC growth. They work together to create a hypoxic and immunosuppressive environment that inhibits antitumor effects and promotes tumor progression and metastasis.^122^ The interaction between CSCs and the TIME remains under active investigation; however, some clues have been found that m^6^A modifications may play an important role in this interaction. m^6^A modification not only influences CSCs and the TIME but also mediates communication between them, and dissecting the detailed role of m^6^A modification will have profound implications in the discovery of novel related targets for tumor therapy.

PD‐L1 signaling is also an essential bridge between CSCs and the TIME. Immune cells produce cytokines to promote PD‐L1 expression in CSCs, while CSCs with a higher PD‐L1 expression induce immune evasion in the TIME.^123^ The m^6^A regulator FTO was identified to participate in this process. FTO enables to upregulate the PD‐L1 expression in colon cancer cells and subsequently promotes immune escape in the TIME.^111^

Integrins family members are known to participate in the signaling transduction between intracellular and extracellular matrix within the TIME and also play a significant role in cancer stemness, progression, and drug resistance.^124^, ^125^ Previous studies have elucidated that integrin‐α_6_ is concentrated on CSCs and maintains the stemness characteristics of CSCs.^126^ Moreover, recent studies have suggested that the m^6^A writer MELLT3 upregulates the expression level of integrin‐α_6_ in BCa cells, facilitating a more malignant phenotype with greater migration and invasion abilities.^127^ Therefore, it is reasonable to speculate that m^6^A modification may also determine the expression level of integrins, which are involved in prosurvival signaling and TIME reprogramming, thus facilitating the stemness features and function of cancer cells.

Taken together, m^6^A regulators are frequently present during the interaction between CSCs and the TIME. Even though the direct regulatory function of m^6^A modifications in this interaction has not yet been uncovered, there are many clues indicating that it plays an important role in regulating a variety of key molecules in this process. The specific communication mechanisms remain elusive, warranting further research to explore the role of m^6^A modification in the CSC‐TIME interplay.

## 4 THERAPEUTIC POTENTIAL OF TARGETING m^6^A REGULATORS

Based on existing research, it is clear that m^6^A regulators play pivotal roles in carcinogenesis, demonstrating great potential in cancer treatment. To date, several relevant studies have explored the therapeutic value of certain m^6^A regulators. FTO inhibitors are the most well‐researched candidates targeting m^6^A modification in cancer therapy, with several FTO inhibitors that increase the m^6^A abundance on RNAs having already been successfully identified. First, FB23 and FE23‐2, other types of small‐molecule FTO inhibitors, are able to bind directly to the FTO active pocket, dealing a fatal blow to the proliferation of AML cells.^128^ In addition, another two FTO inhibitors, CS1 and CS2, have been found to limit the growth and function of LSCs, sensitize them to T‐cell cytotoxicity, and decrease their immune evasion ability.^90^ Another MA2 has also been shown to successfully suppress the phenotype of GSCs and impair tumor progression.^12^ Noticeably, entacapone has been identified as a potential novel FTO inhibitor in metabolic diseases based on the structure virtual screening FDA‐approved drugs. In addition, IGF2BP1 inhibitors have also demonstrated favorable antitumor effects in several malignancies including leukemia, melanoma, and ovarian cancer.^92^, ^129^ Moreover, a small‐molecule ALKBH5 inhibitor, ALK‐04, has been shown to cause a considerable increase in the efficacy of anti‐PD‐1 therapy both in vivo and in vitro.^26^ Further, the simultaneous suppression of METTL3 and METTL14 has also been demonstrated to augment the efficacy of anti‐PD‐1 therapy in colorectal cancer and melanoma, owing to the higher infiltration of CD8+ T cells and massive cytokine release in the TIME.^108^ Additionally, the METTL3 pharmacological inhibitor (STM2457) was also capable of effectively preventing AML growth and improving survival in AML models in preclinical experiments.^88^ Meanwhile, in NSCLC, the resistance to gefitinib caused by METTL3‐mediated autophagy process was reversed by β‐elemene.^130^ Together, these studies indicate that m^6^A regulator inhibitors will be useful in cancer treatment.

On the other hand, a bidirectional dCasRx m^6^A modification editing platform has been constructed, which is composed of nucleus dCasRx and either a reader (METTL3) or an eraser (ALKBH5). This editing platform is capable of regulating the methylation status at specific m^6^A sites in HEK293T and GBS cells, ultimately affecting the expression of target genes and cancer cell proliferation.^131^ This remarkable emerging technology provides a solid foundation for the clinical targeting of m^6^A modification in cancer treatment. Furthermore, some preclinical research also found that depletion of some m^6^A regulators can sensitize tumor cells to chemotherapies, including breast cancer and NK/T cell lymphoma.^132^, ^133^ Nevertheless, further detailed studies are required to realize its potential. Finally, it is notable that m^6^A expression profiles have great potential to differentiate the immune characteristics of patients with tumors, which is likely to accurately guide the application of immunotherapy in clinical.^134^, ^135^

## 5 DISCUSSION AND PERSPECTIVE

Despite still being in the immature stage of exploration, numerous studies have indicated that m^6^A modification and the corresponding regulators orchestrate a range of critical pathological processes in tumorigenesis and development by regulating the epitranscriptome. Notably, m^6^A modification actively participates in the development of CSCs in various tumors, determining their fate and functions to influence tumor progression. Both genetic and pharmacological inhibition of m^6^A regulators enable the suppression of CSCs self‐renewal and growth, thus limiting tumor formation and progression. Moreover, m^6^A modification also remodels the TIME in various aspects, including immune cell regulation, cytokine production, and immune checkpoint expression. Targeting m^6^A regulators is an effective method for sensitizing immune responses in anti‐PD‐1/L1 immunotherapy both in vitro and in vivo, which further profoundly indicates a novel strategy to compensate for the limitations of immunotherapy. The m^6^A modification is a dual regulator in both CSCs and TIME, but its direct function in this interaction has not yet been proposed. However, some clues indicate that it is involved in the deadly teamwork of the CSCs‐TIME. m^6^A regulators collaborate with hypoxic factors, integrins, and PD‐L1 to influence interactions between the CSC and TIME, ultimately promoting the tumor development and progression. It is clear that the CSC‐TIME interplay has an essential function in the therapeutic resistance and unfavorable survival. We believe more relevant research will further reveal the regulate function of m^6^A modification in such crosstalk, which will also be informative for cancer eradication and therapy.

As rapid advancements are made in m^6^A sequencing and detection methods and continuous refinement of m^6^A‐based drugs development is performed, it is promising that m^6^A modification will open a new opportunity for tumor diagnosis and treatment.^136^, ^137^, ^138^, ^139^ Given the central role of m^6^A modification in tumorigenesis and development, it is reasonable to speculate that m^6^A modification possesses significant value in the clinical diagnosis and treatment of cancer. First, m^6^A regulators are promising biomarkers for distinguishing benign from malignant tumors and predicting metastasis, therapy resistance, and recurrence, which may be helpful in early diagnosis and individualized monitoring. In addition to m^6^A regulators, the global m^6^A profile based on blood or tissue may also be a reliable choice as a cancer biomarker for diagnosis, classification, and prognosis, warranting more advanced m^6^A‐seq technologies. Furthermore, it is noteworthy that m^6^A modification is closely related to the most promising immunotherapies, in particular anti‐PD‐1/PD‐L1 monotherapies, and its associated regulators hold great promise for the screening of appropriate immunotherapy candidates to achieve precision medicine. Moreover, the combination of m^6^A‐related biomarkers and other classical biomarkers is likely to spark new ideas for better clinical guidelines.

With regard to cancer treatment, m^6^A modification has demonstrated tremendous potential in various aspects. First, as more studies explore and validate the specificity and side effects of m^6^A regulatory inhibitors and editing platforms, the two m^6^A‐related therapies may function as emerging targeted treatments for tumor eradication. Second, combining m^6^A‐based and mainstream treatments is also an attractive and promising blueprint for the future. These m^6^A‐targeted regimens may compensate for the limitations and deficiencies of other current therapies to some extent. Nevertheless, the timing and sequence of combined regimens are critical during cancer treatment, and more research is needed to investigate and validate optimal decisions with a view to maximizing patient benefit. In addition, it is clear that aberrant m^6^A deposition level and m^6^A regulators expression play pivotal roles in therapeutic resistance mechanisms, such as chemoradiotherapy resistance and immunotherapy unresponsiveness. This may provide a new opportunity to patients with advanced drug‐resistant cancer for whom no medication is currently available, filling the significant gap still remaining in the current field of cancer therapeutics.

## 6 CONCLUSION

In conclusion, we have overviewed the landscape of m^6^A modifications in the cross‐linkage between CSC and TME for the first time, which may bring the possibility of m^6^A modifications as a new therapeutic target for tumor treatment. In addition, we also attempted to point out the direction of m^6^A modifications in the future clinical applications, which can be suggestive for individual therapy and improvement in efficacy of current treatments. Lastly, only the tip of the iceberg has been uncovered regarding the mechanisms related to m^6^A modification in the crosstalk between CSCs and the TIME, and deeper large‐scale studies are warranted for further exploration with a view to opening a new therapeutic avenue in cancer.

## AUTHOR CONTRIBUTIONS

NS and JH designed this study and provided funding support. CQZ, YJL, and ZHZ drafted the manuscript and completed the figures. YJL, GCZ, and PW collected the references and completed the tables. All the authors reviewed and approved the final manuscript.

## CONSENT FOR PUBLICATION

Consent for publication of this paper has been obtained from the authors.

## COMPETING INTERESTS

The authors declare that they have no competing interests.
